# Dog Ownership and Walking: Perceived and Audited Walkability and Activity Correlates

**DOI:** 10.3390/ijerph17041385

**Published:** 2020-02-21

**Authors:** Barbara B. Brown, Wyatt A. Jensen

**Affiliations:** 1 Department of Family & Consumer Studies, University of Utah, Salt Lake City, UT 84112, USA; 2 Utah Department of Health, Salt Lake City, UT 84116, USA; Wyatt.Jensen@gmail.com

**Keywords:** audited walkability, perceived walkability, physical activity, accelerometer, dog ownership, dog walking

## Abstract

Few studies assess dog ownership and walking with both self-reported or perceived and audited or objective walkability and physical activity measures. Across two years, we examined both types of walkability and activity measures for residents living within 2km of a “complete street”—one renovated with light rails, bike lanes, and sidewalks. Audited walkability (Irvine–Minnesota Inventory) was more consistently related to dog ownership and walking groups than perceived walkability (Neighborhood Environment Walkability Scale—Abbreviated). Self-reported leisure walking was much higher (289–383 min per week) among dog walkers than among other groups (100–270 min per week), despite no difference in accelerometer-measured light or moderate-to-vigorous physical activity (MVPA). Furthermore, the most powerful difference between groups involved single-family detached home residence, which was much lower among non-dog-owners (44%) than among non-dog-walkers or dog walkers (81% and 70%, respectively). Given discrepancies across walkability and activity measures, we recommend future use of walkability audits and objectively measured physical activity over the current emphasis on self-report measures. We also urge greater attention to increased densities of housing, which may negatively affect dog ownership levels unless compensating supports for dog ownership and walking are created by public health messaging, dog-friendly policies, and dog-friendly housing and community design.

## 1. Introduction

Dogs provide important sources of psychological and behavioral benefits for their human companions [1,2,3]. Recently, research has focused on the human health benefits from dog walking. However, one of the key published articles that encourages modern dog walking research also underscores the need to focus on benefits to dogs as well [4]. Indeed, finding sufficient humans to adopt needy dogs is a continuing challenge, with the American Society for the Prevention of Cruelty to Animals (ASPCA) reporting that 670,000 of the 3.3 million dogs admitted to shelters are euthanized [5]. We argue that for the well-being of both dogs and humans, it is important to understand what predicts owning a dog as well as what predicts walking a dog. In the current study, we examine demographic, design, and physical activity variables that distinguish non-dog-owners from non-dog-walkers (i.e., dog owners who do not walk their dogs) and dog walkers in neighborhoods near downtown Salt Lake City, Utah. 

### 1.1. Dog Ownership

Among studies in a meta-analysis that examined dog walking, 24% of respondents owned a dog, with a range that varied considerably across studies, from 10% to 57%. Past research shows that both family and housing characteristics are cited as important reasons to get [6] or relinquish [7] a dog. Households with children in the home are more likely to own dogs [8,9]. Living in a single-family detached house also correlates with dog ownership in the UK [10] and the U.S. [11] and Canada [12,13]. Projections are that the U.S. will continue to increase its number of child-free households and elderly households and move toward housing forms with more urbanity attributes, such as greater walkability and proximity to desirable restaurants, shopping, and other urban amenities [14]. Although growing preferences for walkability might encourage dog ownership and walking, it is possible that smaller housing forms, older residents, and fewer children will discourage dog ownership. 

### 1.2. Dog Walking

A meta-analysis of 17 studies of dog owners found that a median of 59% of owners reported walking their dog, although variability was high, ranging from 3% to 80% [15]. Several studies reported more total walking among dog owners than among non-dog-owners, although not all differences were significant. In the California Health Interview Survey, dog owners reported a significant 18.9 more minutes of walking per week than non-pet-owners [16], but in a national consumer study, there was no difference in reported moderate-to-vigorous physical activity (MVPA) between non-dog-owners, non-dog-walkers, and dog walkers (178.3, 198.0, and 200.5 min per week, respectively) [17].

U.S. residents aged 60 or older reported a significant 17.2 more minutes of walking per week than non-dog-owners [18], and those in their 70s reported a significant 35.8 more minutes of dog walking and walking for instrumental purposes than non-dog-owners [19]. In Australia, dog owners walked more than non-dog-owners, with results ranging from a non-significant 6.7 min per week [20] or 18 min per week [4] to a significant 39.4 more walking minutes per week [21]. In Japan, dog owners reported a non-significant 1.9 metabolic equivalents (MET)-h/w more than non-dog-owners [22], but, in a more recent study by the same authors, dog walkers reported a significant 124.0 min per week more walking than non-dog-walkers [23]. In Calgary, dog owners, compared with non-dog-owners, reported 90.3 more minutes in summer and 146.1 more minutes in winter engaged in neighborhood recreational walking [12]. Note that all these studies relied on self-reports, which may have recall errors or biases and overestimates of physical activity relative to objective measures [24,25]. 

Two studies of adult dog walkers that used objective measures revealed greater activity among dog walkers. In Baltimore and Seattle, dog walkers accrued 35 min per day in accelerometer-measured moderate-to-vigorous physical activity, which was a significant 2 min/day more than non-dog-owners and 8 min/day more than non-dog-walkers [26]. In the UK, dog walkers aged 65 and older achieved 1670 more pedometer-measured steps per day than non-dog-walkers [27]. 

These results suggest that sometimes dog walkers, compared with non-dog-walkers, do not achieve additional physical activity and when they do, the amounts vary substantially across studies. When objective measures are used, the differences across dog owner and walker groups are more modest than when self-reports are used. The results also suggest that it may be important to separate dog owners into those who do and do not walk their dogs when examining walking amounts. The varied results also suggest that it is worthwhile to identify neighborhood environmental features that might encourage dog ownership and walking.

### 1.3. Perceived Walkability

When neighborhood walkability is assessed by self-reported walkability, the results have been mixed. For example, San Diego dog walkers, compared with non-dog-walkers, reported better neighborhood aesthetics and more places for walking but did not differ in perceived crime problems in the neighborhood [28]. The bivariate associations were also reduced to insignificance after controlling for 18 other predictors. In contrast, in Calgary, dog walkers reported less street connectivity and pedestrian infrastructure compared with non-dog-owners. Furthermore, a total walkability score was similar for dog walkers and non-dog-owners but higher among non-dog-owners than non-dog-walkers [13]. The three groups did not differ in perceived crime, traffic, or aesthetics, and all the significant bivariate associations were reduced to non-significance after controlling for socio-demographic variables. Perceived safety did not differ for dog walkers vs. non-dog walkers in a four-city study [29]. In Indiana, dog walkers reported better walking environment scales (e.g., interesting paths, grassy open areas) but not perceived crime or traffic compared with non-dog-walkers [30]. The results suggest that perceived walkability often correlates with other predictors in statistical models, reducing significant direct relationships to insignificant multivariate relationships. Furthermore, without assessing features of the neighborhoods, it is difficult to know whether differences found between dog owning and walking groups are due to different perceptions or different features of the neighborhood. 

### 1.4. Audited Walkability

Few studies have examined neighborhood physical features in relation to walking dogs among adults. In Seattle and Baltimore, after sampling neighborhoods by land use variables that represent high (e.g., higher density, mixed use) and low walkable neighborhoods, walkability was higher for non-dog-owners than dog walkers and higher for dog walkers than non-dog-walkers [26]. A similar index predicted whether adolescents who owned dogs walked them [31]. Other studies found that proximity to a dog-supportive park, which provided dog waste disposal bags, related to regular dog walking (i.e., >90 min per week) [32]. Finally, in a dense area of Japan, walkability (e.g., population density, street connectedness, destinations, sidewalks) also predicted fewer dog-owners but more dog walkers among dog owners [33]. These somewhat complicated results underscore the need to examine more environmental walkability audits along with perceived walkability among the three groups of dog owners and walkers.

In sum, the current study examines whether perceived and audited walkability and activity differentiate across three dog owner and walker groups, with separate analyses across two years.

## 2. Materials and Methods

### 2.1. Data

Data for this study are from the Moving Across Places Study (MAPS) in Salt Lake City, Utah, USA. The study was designed to assess whether a new light rail transit line encourages walking to transit. Data were collected before (2012) and after (2013) the completion of the 4.2 km line through the middle of the neighborhood. The study was also able to investigate perceived and audited walkability and how walkability was related to other types of activities, such as biking and use of parks and community centers. Although several parks were located within the neighborhood, none had any dog-specific areas or fencing.

### 2.2. Sample

Blocks within 2 km of the rail line were randomly selected to create the sample. Adults 18 or older were eligible for recruitment if they intended to live there for at least a year, spoke English or Spanish, were not pregnant, and could walk a few blocks. Recruitment was typically conducted door-to-door, although some participants were recruited at community events if otherwise eligible (for additional details, see [34]). Within household selection typically involved the youngest male or oldest female selection metric, which is designed to avoid intrusive household enumeration and assure adequate recruitment of males. If there are two eligible adults in a household, a random number is used to select the participant. If there are ≥3 eligible adults, the youngest male is recruited first, but the eldest female is recruited if he is not available [35]. To be maintained in the sample, participants needed to wear the accelerometer for at least three ten-hour days, a research-based estimate of the minimum number of days needed for reliable activity measurement [36], and have valid GPS data. The authors’ Institutional Review Board approved the informed consent procedures.

### 2.3. Measures

#### 2.3.1. Perceived Walkability

As noted in Jensen et al., the 54-item Neighborhood Environment Walkability Scale—Abbreviated (NEWS-A) [37] provided the starting point for assessing perceived walkability [38]. As recommended, we attempted to replicate the factors identified in a confirmatory factor analysis by Cerin et al. based on 1286 adults from King County, Washington State, which involved 21 items [39]. In order to obtain good model fit, we supplemented with six additional items regarding housing density (n = 1), land use mix-diversity (n = 1), crime safety (n = 3), and walking/cycling facilities (n = 1). Our confirmatory factor analysis yielded six factors from 20 items. We did not employ the perceived street connectivity measure given that it had no parallel factor from the audited walkability scale.

#### 2.3.2. Audited Walkability

We chose the Irvine–Minnesota Inventory because it provided a comprehensive micro-level walkability scale, which focused on each block face—both sides of the street between intersections. As noted from Jensen et al. [38], 40 items were selected by three researchers to provide the best conceptual fit to the factors derived from the analysis of the perceived walkability items. Audited walkability was assessed for quarter-mile (approximately 0.40 km) street network buffers around each participant’s residence. The quarter-mile buffer would be experienced by any resident walking their dog from home and is commonly used in walking research [40,41,42].

#### 2.3.3. Self-Reported Walking

We selected the two walking items from the International Physical Activity Questionnaire (long form, dated 2002) [43]. Participants are asked to recall their ten-minute or longer walks over the last seven days that involved walking to get some place (“walking to travel from place to place”, but excluding leisure, recreation, and exercise walking) and walking for leisure (“recreation, sport, exercise, or leisure”). Scoring protocols sum the occasions by duration products to get a total amount of physical activity in minutes per week, top coding for 180 min per item. Although self-report forms generally show only modest correlation with measured activity, they are widely used and have shown stability over time [43].

#### 2.3.4. Measured Activity

Participants wore Actigraph GT3X+ accelerometers (Actigraph, Pensacola, USA) and GPS units for approximately one week to provide objective measures of physical activity. We used definitions for accelerometer non-wear time and thresholds for light and MVPA based on Troiano et al.’s standards set for the analysis of the National Health and Nutrition Examination Survey (NHANES). Specially, accelerometer non-wear time was defined as 60 min of zero accelerometer counts per minute but allowing for spikes of up to 2 min of 100 counts per minute. Light activity was defined as 100 to 2019 counts per minute and MVPA as 2020 counts per minute or higher [24]. Troiano et al. set the MVPA threshold based on a weighted average of studies published up to that point in time, based on the same accelerometer equipment. Data were standardized to the minute of light or MVPA per 10 h of wear.

#### 2.3.5. Dog Ownership and Walking

Participants were asked if they owned a dog and, if so, if they walked the dog “at least a couple of times a week”. Participants were assigned to one of three categories: non-dog-owners, non-dog- walkers, and dog walkers.

### 2.4. Data Analysis Procedures

Chi-square analyses tested the differences across the three groups of dog ownership and walking for dichotomous variables. One-way ANOVAs with Hochberg GT2 post-hoc tests for unequal cell sizes tested differences for continuous predictor variables [44]. The data showed that little change in dog ownership occurred across one year, so we analyzed both years separately.

A multinomial logistic regression tested multivariate differences across the three groups. Prior to conducting the multinomial regression, ordinary least squares regressions were used to test for multicollinearity. In 2013, the audited measures of access and aesthetics showed multicollinearity (>30 collinearity scores with two variables weighing >0.50 on the variance proportion table) [45]. Therefore, we averaged the two measures into an access/aesthetics composite measure.

## 3. Results

### 3.1. Descriptive Results

In 2012, 63.1% were non-dog-owners, 28% owned and walked their dog, and 9% owned but did not walk their dog. Similar results appeared in 2013, with 60.8% non-dog-owners, 30% owners who walked their dog, and 9.1% who owned but did not walk their dog.

Table 1 shows descriptive data for the socio-demographic, walkability, and activity variables. Housing form was the largest difference across dog owner and walker groups. Only 43% of non-dog-owners lived in detached housing compared with 81% of non-dog-walkers and 70% of dog walkers. In addition, household income was lower among non-dog-owners than among non-dog-walkers. Non-dog-owners were less likely to have children at home than non-dog-walkers. Respondent gender, Hispanic ethnicity, age, and college graduate status were not significantly different across groups.

Among perceived measures, in 2012, non-dog-owners reported better pedestrian infrastructure and more pleasing aesthetics than non-dog-walkers. Although perceived crime differed across the three groups in the one-way ANOVA, the post-hoc tests did not reveal any pairwise differences. Nevertheless, the same pattern as obtained above was evident, namely that non-dog-owners reported the least crime problems and non-dog-walkers reported the most crime problems. In addition, non-dog-owners reported less time spent walking for leisure than dog walkers, and these two groups did not differ in reported time spent walking to get places.

Among audited measures in 2012, non-dog-owners lived in areas that auditors rated to have less accessibility and less crime. Pedestrian infrastructure audited conditions were better among non-dog-owners than among either non-dog-walkers or dog walkers.

In 2013, perceived measures showed that self-reported leisure walking was lowest for non-dog- walkers and significantly lower than that for non-dog-owners, which was significantly lower than that for dog walkers. Although perceived pedestrian infrastructure and aesthetics showed a similar pattern as in 2012, with non-dog-owners perceiving better pedestrian infrastructure and aesthetics than non-dog-walkers, the results were not statistically significant.

Among audited measures in 2013, the combined access/aesthetics score was lower for non-dog-owners than for the two groups of dog owners. Traffic hazards were lower around the homes of non-dog-walkers than for either non-dog-owners or dog walkers. However, crime indicators were higher among non-dog-walkers than for non-dog-owners or dog walkers. Although pedestrian infrastructure, as in 2012, was better for non-dog-owners than for non-dog-walkers, and the one-way differences across the three groups were significant, no pairwise comparisons were significant.

In general, perceived walkability variables were significant 3 out of 10 times and audited walkability variables were significant 7 out of 9 times. Although dog walkers reported more leisure walking than non-dog-walkers in both years, accelerometry-assessed light and MVPA showed no significant differences across the three groups.

### 3.2. Multivariate Differences across The Three Dog Ownership and Walking Groups

Table 2 and Table 3 show the 2012 and 2013 multinomial logistic regression predictors of the dog ownership and walking groups. The non-dog-owner group is always the referent group. In terms of sociodemographic variables, when all the other predictors are controlled, the strongest odds ratios from the list of predictors involve single-family detached home ownership. Detached housing was a predictor that was significant in each analysis, with odds ranging from 2.65 to 3.96 in 2012 and 2.49 to 13.20 in 2013.

In addition, females were less likely to be in the non-dog-walker group than the non-dog-owner group in both 2012 and 2013 for analyses involving perceived walkability and activity. Females were also less likely to be in the non-dog-walker group than the non-dog-owner group in 2012 for the analysis involving audited walkability and activity measures in 2012.

In the multivariate analyses, the only perceived measure that contributed significant amounts of unique variance was self-reported leisure walking. Recall from Table 1 that non-dog-walkers reported the fewest leisure-walking minutes, followed by non-dog-owners, and finally, dog walkers reported the most minutes of leisure walking in 2013.

The significant audited measures, in 2012, show that pedestrian infrastructure, aesthetics, and traffic hazards significantly differentiated dog walkers from non-dog-owners. For these two groups, dog walkers had lower scores on pedestrian infrastructure, higher scores on aesthetics, and higher scores on traffic hazards, consistent with the pattern of results from Table 1. In 2013, non-dog-walkers lived in areas with more audited crime indicators than non-dog-owners.

## 4. Discussion

These results partially replicate past research but also go beyond past research in the ability to present both self-reported and more objective measures and in replicating analyses over time. For the physical activity correlates, the literature review showed that most prior studies utilized self-reported physical activity measures. The results of the current study show that dog walkers report the most minutes walking for leisure and non-dog-walkers report the least number of minutes, with non-dog-owners in between. In fact, dog walkers reported between 57 and 283 more minutes per week of leisure walking than other groups (Table 1). This is most similar to the magnitude of differences that Lail et al. found in Calgary, where dog walkers reported between 90 and 146 more recreational walking minutes per week than non-dog-owners. However, other studies report narrower differences between groups, with a meta-analysis reporting that dog owners report 18 more minutes per week of walking than non-dog-owners [15]. Perhaps contributing to the large differences found in the present study is that the measurement instrument, questions from the International Physical Activity Questionnaire, has been found to yield overestimates of physical activity relative to objective measures [46]. Furthermore, the accelerometer measures showed that dog walkers had no advantage over the other two groups in amounts of objectively measured light or MVPA. It is possible that dog walking may create a sense of more activity. Alternatively, the minutes dog walkers spend walking the dog might reduce the minutes they spend in more instrumental walking to get places, although no such differences across groups were found for self-reported instrumental walking or for total accelerometer measures of light or MVPA (Table 1). Additional studies that use both self-reported and accelerometer-measured physical activity are recommended to explore more fully the fact that differences found in self-reports are not reflected in accelerometer measures. The use of experience sampling methodologies might allow for stronger tests of correspondence between self-reported dog walks and accelerometer-detected bouts of activity.

This study also provided both trained-rater-assessed and self-reported walkability in relationship to dog owning and walking. Here, the findings partially replicate the two studies of audited walkability among adults. Coleman et al. assessed high- and low-walkability neighborhoods in Seattle and Baltimore, and Koohsari et al. assessed high- and low-walkability cities in Japan [26,33]. Coleman et al. assessed walkability features of density, street connectivity, and land use mix, which were combined into an index, and Koohsari et al. assessed similar walkability features, which were analyzed separately. Although their technique of sampling very diverse geographic areas should facilitate the search for walkability differences compared with the current study of a contiguous geographic area, a similar pattern of results was found. Specifically, all three studies found objectively measured walkability advantages where non-dog-owners lived. Similarly, the current study found that non-dog-owners lived in areas with better pedestrian infrastructure, lower traffic hazards, and fewer indicators of crime than one of the dog-owning groups. Among dog owners, Coleman et al. also found greater walkability for dog walkers over non-dog walkers. Koohsari et al. found that this was true for the street integration measure of walkability. The current study found that dog walkers lived in areas of lower crime but worse traffic hazards than non-dog walkers. As discussed below, the somewhat consistent finding of walkability benefits for non-dog owners might reflect constrained housing and neighborhood options for dog owners.

In terms of perceived walkability, the results of the current study echo the findings of other studies that infrequently found strong links between dog walking or ownership and better perceived walkability. In San Diego, Hoerster et al. found that dog walkers reported more places for walking and pleasant aesthetics than non-dog walkers but these results were reduced to non-significance when controls were added to the model; perceived crime did not differ [28]. In Calgary, non-dog-owners perceived greater street connectivity and pedestrian infrastructure than dog walkers and more total walkability than non-dog walkers; however, these univariate differences were not tested in a multivariate model [13]. In Portland, dog walkers perceived more neighborhood problems, including traffic and crime problems, than non-dog walkers [29]. However, this finding was not replicated in San Diego, Nashville, or Perth. These studies of perceived walkability suggest that dog walkers do not have an especially strong or consistent sense of walkability. The current results reflect those from Hoerster et al. in that significant univariate results disappear when control variables are added to the multivariate equations. The current study is similar to the Calgary study in that the univariate perceived walkability differences are more positive for non-dog-owners than for dog owners. As Christian et al. suggest, dog walkers may have greater knowledge of their neighborhood conditions, including poor walkability conditions [29].

When comparing how audited and perceived walkability measures relate to dog owning and walking, the results suggest that perceived measures of walkability did not serve the same function in supporting dog ownership and walking as did audited measures of the physical environment. From the univariate relationships in Table 1, the audited walkability measures were more consistently related to differences among the dog owning and walking groups than were the perceived measures. Across the two years of the study period, the audited measures had significant relationships to the three groups of dog owners and walkers in 7 out of 9 analyses; for perceived measures, only 3 out of 10 measures showed a significant direct relationship. Just as the Hoerster et al. study found, most of the significant univariate relationships became insignificant after controlling for all other predictors in the multivariate models; none of the perceived measures retained significance, and only 4 out of the 18 audited measures retained significance. This suggests that there is substantial overlap among sociodemographic, walkability, and activity variables in the model.

In fact, the most consistent predictor across all multivariate models is whether the participant lived in single-family detached housing. Only 42% of non-dog-owners lived in detached housing compared with 70% of dog walkers and 81% of non-dog-walkers. Similarly, McCormack et al. reported detached housing for 69.8% of non-dog-owners, 86.7% of non-dog walkers, and 86.9% of dog walkers [13]. Lail et al. reported detached or semidetached housing for 68.4% of non-dog-owners and 91.3% of dog owners (walkers and non-walkers combined) [12]. However, most studies cited in this article did not assess the specific role of housing form in dog ownership and walking. In future research, it would be useful to ask dog owners if they believe their home or yard space gives the dog sufficient activity space, given that non-dog-walkers were most likely to live in a detached home. Given the striking differences found here favoring single-family detached home forms among dog owners, we recommend that future researchers include housing type in their models.

As the world urbanizes, denser housing options are needed to provide affordability, environmental benefits, and healthy walking opportunities [47]. One unintended consequence may be that dog ownership becomes more challenging if dogs are seen as less suitable to higher density housing and community designs. Research shows that one of the main reasons for giving up a dog is landlord and housing problems [48]; in dense areas of Hong Kong, dog owners report disputes over the proper length of a leash or whether dogs are welcome in high-rise elevators [49]. Dog owners who do not have private housing report that they may accept less desirable housing and neighborhoods in order to find a rental unit that will accept pets [50]; this might explain why non-dog owners were living in more walkable areas in this study as well as others. It is possible that community design might compensate by providing dog-friendly parks or policies. For example, dog parks might encourage more use [29], or “loaner dogs” might accommodate the needs of those who cannot keep dogs [51]. Other suggestions relate to getting more physical activity with dogs, which might involve dog walking groups, more mass media campaigns, and supporting national policy statements that encourage dog walking [32,51,52,53].

The current study balances strengths of objective measures for audits and accelerometry against inevitable limitations. We did not choose our sample area to vary along dimensions of walkability, given that the goal of the underlying project was to investigate one bounded geographic area. In addition, our self-reported walking did not specify walking with dogs in the neighborhood, although most dog walking is done in one’s neighborhood [52]. Nor did our measures assess who else in the household might have walked the dog. Non-dog walkers reported the highest percentage of children in the home, suggesting that children might have taken the dog for walks. One study found that 62% of 12- to 17-year-old children in dog-owning households report walking the dog at least weekly [31]. Perceived walkability measures also typically include fewer survey items compared with more numerous specific details represented in environmental audits; thus, comparative analyses are designed to be conceptually similar but without identical items. The one-year time interval between our study phases also did not allow sufficient changes in dog ownership to track longitudinal changes in physical activity or walkability measures. However, we were able to demonstrate that of the nine significant effects found in 2012, five of them were significant again in 2013, suggesting some replicability of effects.

Given that our study demonstrated that residents said they achieved more leisure walking time when they were dog walkers but that accelerometer measures did not confirm these results, it is important to consider possible reasons. Dog walks are complex events in which the amount of physical activity may not be recalled accurately because the activity is interwoven with other experiences. A dog walk can become a “dog-stand” while chatting with neighbors or a “dog-sit” while owners appreciate watching the sunset or dogs cavorting together. Humans may overestimate how much physical activity they accrue on dog walks, given the natural diversions into more sedentary activity along the way. Dog walkers who choose to set specific goals for steps or physical activity minutes during dog walks may want to verify their activity amounts against objective measures, such as pedometers or activity watches. If activity goals fall short, the dog and owner might enjoy extending their walk, or the owner may compensate with more time on the exercise bike back home. However, the advice to consult activity feedback might backfire, if, as a reviewer suggested, the feedback transforms the complex enjoyable walk into a less enjoyable, externally motivated quest by humans for more steps. We acknowledge that there are many parts of a dog walk beyond physical activity that make them healthy for dogs and their owners—pleasurable routines, neighborly interactions, reduced stress, psychological well-being, and enhanced bonds [1,3,53]. Future research is needed to determine how the varied events on a dog walk, including gauging one’s physical activity, may foster or thwart the ability to sustain a healthy dog walking routine.

## 5. Conclusions

The current study revealed a number of relationships between walkability and physical activity among our three groups: non-dog-owners, non-dog-walkers, and dog walkers. More audited walkability than perceived walkability indicators were related to dog owning and walking groups. Furthermore, dog walkers reported high levels of leisure walking, but these high levels were not corroborated by objective accelerometer measures. We encourage future researchers to replicate these results. If replicable, there needs to be more public health efforts to assess the effects of encouraging dog walkers to track their activity using step or activity counters, to see if this aids in physical activity goals and dog walking enjoyment. These monitoring efforts could be complemented by housing policy and community design efforts to make dog ownership and walking an easier choice than may be experienced by many residents who live in dwellings that are not single-family detached homes. In addition, techniques that encourage physical activity to become habitual, such as providing attractive dog parks or encouraging group walks, may be needed to turn occasional walks into measurable physical activity increases.

## Figures and Tables

**Table 1:** Descriptive statistics (% or M and SD) among non-dog-owners (NDO), non-dog-walkers (NDW), and dog walkers (DW) with Chi-square or one-way ANOVA results (n = 536).

Variables	NDO (a)	NDW (b)	DW (c)	*p*	Post-hoc
Socio-demographics, 2012:					
Female (%)	51.48	39.58	52.67	0.26	
Hispanic (%)	23.81	29.79	24.67	0.67	
Children at home	37.28	56.25	44.00	0.03	a < b
College graduate	35.21	43.75	38.26	0.47	
Detached housing	43.49	81.25	70.00	0.01	a < b = c
Age (in years)	41.57 (14.97)	41.50 (15.51)	42.12 (14.14)	0.93	
Household income ($1000s)	38.57 (30.28)	53.13 (40.77)	44.60 (31.05)	0.01	a < b
Perceived measures, 2012:					
Accessibility	−0.01 (0.25)	−0.05 (0.24)	0.00 (0.26)	0.43	
Pedestrian infrastructure	0.03 (0.36)	−0.11 (0.36)	−0.04 (0.37)	0.01	a > b
Aesthetics	0.00 (0.65)	−0.31 (0.63)	−0.09 (0.64)	0.01	a > b
Traffic hazards	−0.01 (0.27)	0.07 (0.28)	0.04 (0.28)	0.07	
Crime	−0.07 (0.68)	0.15 (0.75)	0.09 (0.73)	0.02	
Walk to get places (min/w)	311.27 (374.55)	242.08 (357.49)	326.20 (422.06)	0.42	
Walk for leisure (min/w)	233.27 (339.61)	151.46 (247.62)	289.83 (386.34)	0.04	b < c
Audited measures, 2012:					
Accessibility	3.38 (0.48)	3.57 (0.37)	3.48 (0.40)	0.01	a < b
Pedestrian infrastructure	1.73 (0.75)	1.43 (0.56)	1.53 (0.64)	0.01	a > b = c
Aesthetics	5.28 (1.82)	5.36 (1.41)	5.46 (1.54)	0.56	
Traffic hazards	4.62 (0.89)	4.37 (0.95)	4.63 (0.94)	0.17	
Crime	1.67 (0.49)	1.96 (0.54)	1.83 (0.53)	0.01	a < b = c
Light activity (min/10 h)	210.21 (60.90)	218.92 (64.45)	216.76 (52.45)	0.40	
Moderate-to-vigorous (min/10 h)	20.59 (18.08)	20.25 (19.29)	19.97 (17.05)	0.94	
Perceived measures, 2013:					
Accessibility	0.01 (0.22)	−0.01 (0.22)	−0.01 (0.23)	0.63	
Pedestrian infrastructure	0.02 (0.39)	−0.10 (0.39)	0.00 (0.42)	0.18	
Aesthetics	0.01 (0.63)	−0.17 (0.69)	0.03 (0.66)	0.16	
Traffic hazards	−0.02 (0.25)	0.07 (0.27)	0.01 (0.26)	0.08	
Crime	−0.04 (0.72)	0.17 (0.76)	0.03 (0.77)	0.14	
Walk to get places (min/w)	300.08 (389.98)	253.13 (358.72)	281.24 (382.86)	0.69	
Walk for leisure (min/w)	269.74 (370.29)	100.21 (245.49)	383.48 (445.86)	0.00	b < a < c
Audited measures, 2013:					
Access/aesthetics	4.70 (1.05)	5.14 (0.55)	4.94 (0.90)	0.01	a < b = c
Pedestrian infrastructure	2.04 (0.79)	1.82 (0.53)	1.90 (0.61)	0.04	
Traffic hazards	4.95 (0.84)	4.47 (0.78)	4.81 (0.84)	0.01	b < a = c
Crime	2.61 (0.73)	2.95 (0.61)	2.65 (0.74)	0.01	a = c < b
Light activity (min/10 h)	215.57 (64.78)	226.87 (67.25)	221.41 (53.34)	0.37	
Moderate-to-vigorous (min/10 h)	20.70 (17.70)	20.54 (23.58)	21.75 (18.31)	0.83	

Note: Post-hoc results are reported in the final column when *p* < 0.05.

**Table 2:** Perceived and audited walkability and activity in 2012: multinomial analysis of dog owning and walking groups. MVPA: moderate-to-vigorous physical activity.

	B	SE	P	Odds	Lower CI	Upper CI
**Perceived measures, 2012**						
Non-dog walkers, intercept	−3.29	0.68	0.00			
Female	−0.69	0.35	**0.05**	0.50	0.25	0.98
Detached housing	1.38	0.41	**0.00**	3.96	1.77	8.90
Accessibility	−0.16	0.74	0.83	0.85	0.20	3.64
Pedestrian infrastructure	−0.26	0.62	0.68	0.77	0.23	2.62
Aesthetics	−0.52	0.38	0.16	0.59	0.28	1.24
Traffic hazards	−0.52	0.92	0.57	0.59	0.10	3.61
Crime	0.13	0.32	0.67	1.14	0.61	2.12
Walk to get places (min/w)	0.00	0.00	0.59	1.00	1.00	1.00
Walk for leisure (min/w)	0.00	0.00	0.35	1.00	1.00	1.00
Dog walkers, intercept	−1.79	0.40	0.00			
Female	0.00	0.22	0.98	1.00	0.66	1.54
Detached housing	1.04	0.23	**0.00**	2.84	1.82	4.42
Accessibility	0.53	0.49	0.28	1.70	0.65	4.45
Pedestrian infrastructure	−0.65	0.39	0.10	0.52	0.24	1.13
Aesthetics	0.11	0.23	0.65	1.11	0.71	1.75
Traffic hazards	0.28	0.59	0.64	1.32	0.41	4.23
Crime	0.09	0.20	0.67	1.09	0.73	1.62
Walk to get places (min/w)	0.00	0.00	0.40	1.00	1.00	1.00
Walk for leisure (min/w)	0.00	0.00	0.10	1.00	1.00	1.00
**Audited measures, 2012**						
Non-dog walkers, intercept	−5.14	2.33	0.03			
Female	−0.75	0.36	**0.04**	0.47	0.23	0.96
Detached housing	1.25	0.43	**0.00**	3.50	1.51	8.09
Accessibility	0.25	0.55	0.65	1.28	0.44	3.76
Pedestrian infrastructure	−0.29	0.37	0.43	0.75	0.36	1.54
Aesthetics	0.05	0.15	0.75	1.05	0.79	1.39
Traffic hazards	−0.04	0.24	0.88	0.96	0.60	1.55
Crime	0.73	0.40	0.07	2.08	0.95	4.54
Light activity (min/10 h)	0.00	0.00	0.53	1.00	1.00	1.01
MVPA (min/10 h)	0.00	0.01	0.76	1.00	0.98	1.02
Dog walkers, intercept	−4.79	1.30	0.00			
Female	−0.06	0.22	0.77	0.94	0.61	1.45
Detached housing	0.97	0.24	**0.00**	2.65	1.66	4.22
Accessibility	0.00	0.28	1.00	1.00	0.57	1.74
Pedestrian infrastructure	−0.44	0.21	**0.04**	0.64	0.42	0.98
Aesthetics	0.22	0.09	**0.01**	1.25	1.04	1.49
Traffic hazards	0.39	0.16	**0.01**	1.48	1.09	2.00
Crime	0.39	0.27	0.16	1.47	0.86	2.52
Light activity (min/10 h)	0.00	0.00	0.54	1.00	1.00	1.00
MVPA (min/10 h)	0.00	0.01	0.75	1.00	0.99	1.01

Note: Non-dog-owners = reference category. Controlled for gender, Hispanic ethnicity, children in home, college graduate status, age, and household income. Bold numbers are significant, *p* < 0.05.

**Table 3:** Perceived and audited walkability and activity in 2013: multinomial analysis of dog owning and walking groups.

	B	SE	*p*	Odds	Lower CI	Upper CI
**Perceived measures, 2013**						
Non-dog walkers, intercept	−4.72	0.82	0.00			
Female	−0.70	0.36	**0.05**	0.50	0.24	1.01
Detached housing	2.58	0.56	**0.00**	13.20	4.45	39.21
Accessibility	0.99	0.94	0.29	2.70	0.43	17.02
Pedestrian infrastructure	−0.49	0.56	0.39	0.61	0.20	1.85
Aesthetics	0.05	0.36	0.89	1.05	0.52	2.14
Traffic hazards	1.16	1.15	0.31	3.19	0.34	30.21
Crime	−0.14	0.35	0.69	0.87	0.44	1.72
Walk to get places (min/w)	0.00	0.00	0.36	1.00	1.00	1.00
Walk for leisure (min/w)	0.00	0.00	**0.00**	1.00	1.00	1.00
Dog walkers, intercept	−1.12	0.39	0.00			
Female	−0.10	0.22	0.63	0.90	0.59	1.37
Detached housing	1.03	0.22	**0.00**	2.80	1.81	4.32
Accessibility	−0.61	0.55	0.26	0.54	0.18	1.59
Pedestrian infrastructure	−0.21	0.35	0.54	0.81	0.41	1.60
Aesthetics	0.37	0.23	0.10	1.45	0.93	2.25
Traffic hazards	0.45	0.69	0.52	1.56	0.40	6.10
Crime	0.01	0.21	0.97	1.01	0.66	1.53
Walk to get places (min/w)	0.00	0.00	0.13	1.00	1.00	1.00
Walk for leisure (min/w)	0.00	0.00	**0.00**	1.00	1.00	1.00
**Audited measures, 2013**						
Non-dog-walkers, intercept	−4.96	2.78	0.07			
Female	−0.59	0.37	0.11	0.56	0.27	1.15
Detached housing	2.13	0.57	**0.00**	8.46	2.79	25.66
Access/aesthetics	0.19	0.30	0.54	1.21	0.66	2.19
Pedestrian infrastructure	−0.44	0.34	0.19	0.64	0.33	1.24
Traffic hazards	−0.42	0.28	0.14	0.66	0.38	1.15
Crime	0.73	0.31	0.02	2.07	1.12	3.81
Light activity (min/10 h)	0.00	0.00	0.65	1.00	1.00	1.01
MVPA (min/10 h)	0.00	0.01	0.87	1.00	0.98	1.02
Dog walkers, intercept	−2.43	1.38	0.08			
Female	0.03	0.22	0.91	1.03	0.67	1.58
Detached housing	0.91	0.23	**0.00**	2.49	1.57	3.93
Access/aesthetics	0.17	0.14	0.23	1.18	0.90	1.56
Pedestrian infrastructure	−0.29	0.16	0.08	0.75	0.54	1.03
Traffic hazards	0.15	0.16	0.34	1.16	0.85	1.58
Crime	0.02	0.15	0.89	1.02	0.76	1.37
Light activity (min/10 h)	0.00	0.00	0.79	1.00	1.00	1.00
MVPA. (min/10 h)	0.01	0.01	0.30	1.01	0.99	1.02

Note: Non-dog-owners = reference category. Controlled for gender, Hispanic ethnicity, children in home, college graduate status, age, and household income. Bold numbers are significant, *p* < 0.05.
